# The mobile sleep medicine model in neurologic practice: Rationale and application

**DOI:** 10.3389/fneur.2022.1032463

**Published:** 2022-10-28

**Authors:** Mark I. Boulos, Luqi Chi, Oleg Y. Chernyshev

**Affiliations:** ^1^Hurvitz Brain Sciences Research Program, Sunnybrook Research Institute, Sunnybrook Health Sciences Centre, Toronto, ON, Canada; ^2^Division of Neurology, Department of Medicine, University of Toronto, Toronto, ON, Canada; ^3^Sleep Laboratory, Sunnybrook Health Sciences Centre, Toronto, ON, Canada; ^4^Washington University School of Medicine, St. Louis, MO, United States; ^5^Sleep Medicine Division, Department of Neurology, Louisiana State University Health Sciences Center, Shreveport, LA, United States; ^6^Ochsner LSU Health Sleep Medicine Center, Shreveport, LA, United States

**Keywords:** stroke, neuromuscular conditions, ambulatory sleep testing, home sleep apnea test (HSAT), screening, mobile sleep medicine, sleep-disordered breathing, cognitive impairment

## Abstract

**Background:**

Undiagnosed obstructive sleep apnea (OSA) is prevalent in neurological practice and significantly contributes to morbidity and mortality. OSA is prevalent in US adults and causes poor quality sleep and significant neurocognitive, cardiovascular, and cerebrovascular impairments. Timely treatment of OSA reduces cardio-cerebrovascular risks and improves quality of life. However, most of the US population has limited systematic access to sleep medicine care despite its clinical significance.

**Focus:**

We discuss the importance of systematic screening, testing, and best-practice management of OSA and hypoventilation/hypoxemia syndromes (HHS) in patients with stroke, neurocognitive impairment, and neuromuscular conditions. This review aims to introduce and describe a novel integrated Mobile Sleep Medicine (iMSM) care model and provide the rationale for using an iMSM in general neurological practice to assist with systematic screening, testing and best-practice management of OSA, HHS, and potentially other sleep conditions.

**Key points:**

The iMSM is an innovative, patient-centered, clinical outcome-based program that uses a Mobile Sleep Medicine Unit—a “sleep lab on wheels”—designed to improve access to OSA management and sleep care at all levels of health care system. The protocol for the iMSM care model includes three levels of operations to provide effective and efficient OSA screening, timely testing/treatment plans, and coordination of further sleep medicine care follow-up. The iMSM care model prioritizes effective, efficient, and patient-centered sleep medicine care; therefore, all parties and segments of care that receive and provide clinical sleep medicine services may benefit from adopting this innovative approach.

## Introduction

Undiagnosed and untreated sleep-related breathing disorders (SDB) are prevalent in neurological practice and contribute significantly to the development of morbidity and mortality (1–10). In the US, 70% of adults report obtaining insufficient/“unprotected” sleep at least one night a month, and 11% report obtaining insufficient sleep every night. Sleep-related problems are estimated to affect 50–70 million Americans of all ages and socioeconomic classes (11, 12).

Approximately 25 million US adults suffer from obstructive sleep apnea (OSA)—a leading sleep-disruptive force that breaks down sleep protection and causes insufficient sleep, significant neurocognitive, cardiovascular, and cerebrovascular (CCV) impairments in humans (13). The timely treatment of OSA with positive airway pressure (PAP) therapy protects human sleep, reduces cardio-cerebrovascular risks and improves the quality of human life (4–10, 14).

Currently, 80% of the general US population and about 36 million annually hospitalized patients have limited systematic access to sleep medicine care despite alarming statistics affirming the significant prevalence of OSA in vulnerable populations.

While the prevalence of OSA in an unselected adult population is generally estimated at ~5% (4, 15, 16), the prevalence of OSA in some medical and neurological populations is significantly elevated, ranging from 10 to 82% in patients with epilepsy (1–3), from 50 to 94% in patients with stroke and transient ischemic attack (TIA) (4–10), from 11 to 82% in patients with arterial hypertension (HTN) (17, 18), from 30 to 40% in patients with the acute coronary syndrome (ACS) and myocardial infarction (MI) (4, 14), from 3 to 49% in patients with atrial fibrillation (19–22), from 10 to 43% in patients with congestive heart failure (CHF) (4, 23–25), 23% in patients with traumatic brain injury (TBI) (26), from 21 to 38% in patients with multiple sclerosis (MS) (27, 28), from 20 to 56% in patients with Parkinson's disease (PD) (29–34), 40% in patients with Alzheimer's dementia (35), from 11 to 36% in patients with neuromuscular conditions (36), and 17 to 76% in patients with amyotrophic lateral sclerosis (ALS) (37, 38).

Timely delivered PAP therapy appears to have beneficial effects on patients with OSA in the general population (39, 40), ischemic stroke and TIA (41–43), epilepsy (44–46), HTN ACS/MI (47–50), atrial fibrillation (51–53), CHF (54–56), TBI (26), multiple sclerosis (27), PD (57), Alzheimer's dementia (35, 58–60) and neuromuscular conditions (61, 62), and ALS (16, 62–65).

Timely delivered non-invasive airway pressure therapy (NIPAP) appears to produce beneficial effects on patients with sleep-related hypoventilation/hypoxemia syndromes (HHS) in neuromuscular disorders (61, 62), obesity hypoventilation (66), and ALS (16, 62–65).

The goals of this review are to (1) introduce and describe a novel, integrated Mobile Sleep Medicine care model (iMSM); (2) provide the rationale for the implementation of iMSM in general neurological practice to assist with systematic screening, testing, and the best-practice management of the most common SDB (OSA and HHS) in patients with stroke, neurocognitive impairment, and neuromuscular conditions (67).

## The rationale for implementation of iMSM in general neurological practice

Here, we discuss the importance of systematic screening, testing, and best-practice management for OSA and HHS in patients with (1) stroke, (2) neurocognitive impairment, and (3) neuromuscular conditions.

### Sleep-disordered breathing in patients with stroke

OSA causes intermittent pauses in breathing (apneas) during sleep, exposing the cardiovascular system to recurrent physiological stressors that activate the sympathetic nervous system, raise blood pressure, and impair vascular endothelial function (68). Such physiological alterations closely link OSA with vascular disease, and OSA is an independent risk factor for HTN (69), atrial fibrillation (70), MI (71), stroke (72), and mortality (73).

#### OSA is prevalent after stroke/TIA

OSA occurs in more than 70% of stroke/TIA survivors (6), and even 3 years after stroke, the prevalence of OSA remains just as high (74), suggesting that screening for OSA at any point after a cerebrovascular event may provide clinical benefit. Rates of OSA do not vary according to the method of detection used (e.g., polysomnography vs. portable sleep equipment) (6).

#### OSA negatively impacts outcomes post-stroke

Left untreated, moderate-to-severe post-stroke OSA garners a three-fold increase in mortality (41) and a five-fold increase in recurrent vascular events (75). Moreover, a diagnosis of OSA is an independent predictor of worse functional outcomes at hospital discharge (76, 77) and is associated with longer hospitalization in the rehabilitation setting (78).

#### Post-stroke treatment of OSA improves outcomes

Treatment of post-stroke OSA using continuous positive airway pressure (CPAP) in randomized trials has been shown to improve functional, and motor outcomes (79–84), increase quality of life (85), reduce daytime sleepiness (81, 85), improve mood (86), and enhance cognition (59, 87). In observational studies, compliance with CPAP has been shown to reduce incident vascular events (75) and mortality (41). Outside the stroke/TIA setting, RCTs have demonstrated beneficial effects of CPAP for blood pressure (88) and lipid control (89).

#### Clinicians do not routinely screen for OSA after stroke or TIA

Despite its high prevalence and detrimental effect on health, OSA remains underdiagnosed and undertreated after stroke/TIA (90). Detection of OSA after stroke/TIA is challenging because OSA presents atypically in patients with cerebrovascular disease (e.g., the absence of excessive daytime sleepiness or obesity) (91, 92), not surprisingly, stroke clinicians infrequently refer patients for sleep-related investigations (90). Since commonly used screening questionnaires show poor correlations with polysomnography findings in stroke patients (93), objective measures are best suited to accurately diagnose OSA.

#### In traditional sleep medicine testing model the lack of availability of in-laboratory polysomnography (“Gold Standard”) is a major barrier to efficiency screening for OSA in hospital settings

Technologist-monitored, in-laboratory sleep studies (polysomnography or PSG) are the “Gold Standard” for diagnosing OSA, but limited availability of polysomnography and lengthy wait times frequently prohibit timely evaluations (90, 94). In addition, some patients are unwilling to spend a night in a sleep laboratory. Finally, polysomnography involves costly equipment, an on-site technologist, and high healthcare expenditures.

#### OSA can be accurately detected using a simple, portable sleep monitoring (“Silver Standard”)

These devices are much less expensive, more accessible, and more convenient and demonstrate good diagnostic performance compared with in-laboratory polysomnography in adult patients with a high pretest probability of moderate to severe obstructive sleep apnea (e.g., the post-stroke/TIA population) (95, 96).

#### Home/hospital sleep apnea testing (HSAT; “Silver Standard”) has been shown to improve outcomes after stroke/TIA

In a randomized controlled trial involving 250 consecutively recruited stroke/TIA patients, patients were randomized to undergo home/hospital sleep apnea testing (HSAT) vs. in-laboratory polysomnography. Those randomized to HSAT had higher rates of OSA diagnosis and treatment, reduced daytime sleepiness, and improved functional outcomes. Moreover, a cost-effectiveness analysis, including the cost of initial diagnostic tests, broken/lost equipment, etc., revealed that HSAT was economically attractive for detecting OSA compared with in-laboratory sleep testing (97). Overall, this study suggested that ambulatory “Silver Standard” approach to sleep testing may improve clinical outcomes in stroke/TIA patients. The cost-effectiveness of an ambulatory “Silver Standard” approach to sleep testing *via* the mobile sleep unit would likely extend to other clinical populations at high risk for OSA.

### Sleep disorder breathing in Alzheimer's disease/dementia

#### Cognitive impairment has a devastating impact on society

Approximately 747,000 Canadians live with Alzheimer's disease or another form of dementia (98), and the associated care costs ~$33 billion annually (98). **Alzheimer's disease (AD)** is the leading cause of dementia worldwide (99). **Vascular cognitive impairment (VCI)** is the clinical syndrome in which cognitive impairment, encompassing mild cognitive impairment and dementia, can be attributed to vascular disease such as clinically overt stroke and/or silent brain infarction (100, 101). Vascular brain injury accounts for up to 33% of dementia risk according to autopsy studies (102) and frequently co-exists with AD (103), making VCI a significant public health issue (101). **Mild cognitive impairment (MCI)** causes cognitive problems that do not interfere with everyday life. However, amnestic MCI increases the risk of developing AD (98).

#### OSA is closely linked with AD and VCI

OSA gives rise to physiological alterations that contribute to the cognitive impairment seen in both AD and VCI, such as transient sympathetic activation, systemic inflammation, endothelial dysfunction in the vasculature of the brain (104), and impaired sleep-dependent memory consolidation through sleep fragmentation (105). Sleep fragmentation from OSA can also lead to impaired glymphatic and vascular drainage of amyloid (106, 107), which is postulated to contribute to the development of AD (108). Furthermore, OSA is strongly associated with cerebrovascular disease and is an independent risk factor for high blood pressure (69), overt (73), and covert stroke (109).

#### OSA is an independent risk factor for AD, VCI, and MCI

In elderly patients without dementia at baseline, the presence of OSA is an independent risk factor for the development of MCI or dementia (110), and a meta-analysis suggested that OSA is an important modifiable risk factor for dementia and other cognitive impairment (111). Furthermore, given the close association of neurodegeneration with vascular disease, treatment of OSA—a well-established vascular risk factor (68)—may also have important implications for brain health even beyond the potentially beneficial effects on cognition and daily function.

#### Obstructive sleep apnea is prevalent in AD/VCI

More than 70% of patients with VCI endorse symptoms consistent with OSA (112). Moreover, prior work has demonstrated that nearly 90% of patients with Alzheimer's disease have obstructive sleep apnea when objectively tested (113). Again, rates of OSA do not vary with the method of detection (i.e. “Gold Standard” vs. “Silver Standard”) (6).

#### Treatment of OSA using CPAP improves cognition

OSA is treated with CPAP, which provides mild air pressure to maintain airway patency during sleep. A review of five systematic reviews and meta-analyses concluded that treatment with CPAP improved attention/vigilance, executive dysfunction, memory, and global cognitive functioning in non-demented individuals (114). A more recent systematic review demonstrated a protective effect that treatment with CPAP has on MCI and AD incidence (115). Several studies have suggested that treating OSA using CPAP in AD patients may slow the rate of cognitive decline (58, 59, 116). In the only randomized controlled trial examining subjects with VCI and OSA, CPAP-treated patients showed improved attention and executive functioning compared to controls who did not receive CPAP (87).

#### Clinicians do not routinely screen for OSA in patients with AD/VCI

Despite its high prevalence and negative impact on cognition, OSA remains underdiagnosed and undertreated.

#### Use of polysomnography within traditional sleep medicine testing model is associated with many barriers

Technologist-monitored, overnight, level 1 PSG (“**Gold Standard**”) is the current standard tool for diagnosing sleep disorders, but high costs, lengthy wait times, and patient unwillingness to spend a night in a sleep laboratory frequently prohibit timely assessments (117). Moreover, the traditional sleep medicine testing model is particularly inconvenient for patients with cognitive impairment, who may depend on others for care and may require a familiar environment to sleep and avoid delirium.

HSAT (“Silver Standard”), has been extensively validated against in-laboratory PSG for detecting obstructive sleep apnea (95, 96, 118). In addition, an unattended sleep study (“**Silver Standard**”) may be potentially less expensive and more accessible.

#### HSAT is feasible for use in patients with AD/MCI

Our prior work has demonstrated that the use of unattended sleep study (“**Silver Standard**”) is feasible in patients with AD/MCI/VCI; >85% of patients who attempted to use unattended sleep study (“**Silver Standard**”) were able to obtain analyzable data (119).

### Sleep-disordered breathing in neuromuscular disorders

In patients with progressive neuromuscular disease (NMD), respiratory failure caused by respiratory muscle weakness is the most common cause of death. Respiratory muscle weakness, changes in chest wall mechanics, and difficulty with airway secretion clearance leads to ineffective alveolar ventilation and both acute and chronic respiratory failure (120).

#### Physiological change in sleep

During sleep, minute ventilation decreases, muscle activity alters, respiratory workload increases (121), ventilatory response to hypoxemia and hypercapnia declines (122), and upper airway resistance increases (123). In REM sleep, skeletal muscle tone is abolished, while the diaphragm is relatively spared (121). Patients with NMD cannot compensate for physiological changes in sleep, and inadequate alveolar ventilation may first occur during REM sleep. As the disease progresses, hypoventilation may extend to non-REM sleep, and eventually, daytime hypercapnia may occur.

#### Prevalence of SDB

The prevalence of SDB is high in most NMDs, and chronic respiratory failure occurs with disease progression. A prior study using a home PSG sleep study without CO_2_ monitoring showed a high prevalence of SDB in a group of chronic neuromuscular disorders. The prevalence of SDB with respiratory disturbance index (RDI) >15/h was 42%, higher than the general population. Respiratory events were primarily hypopneas, and 23% of patients had nocturnal hypoxemia (124). Patients with NMD usually have a normal ventilatory drive (125). Using EMG to monitor the activity of respiratory muscles during PSG in patients with NMD revealed that hypopneas that happened in REM sleep were mostly “central” in nature due to reduced muscle activity, and that nocturnal hypoxemia was inversely correlated with diaphragm strength (126). Studies have confirmed that nocturnal hypoxemia and hypercapnia in REM sleep are common in patients with NMD (127, 128).

#### Non-invasive ventilation (NIV) significantly benefits the quality of life, survival, and respiratory status in different NMD types

Studies have provided evidence that NIV significantly prolonged survival in patients with ALS (129–132). Early initiation of NIV slowed the rate of forced vital capacity (FVC) decline (131) and improved survival (131, 133). Berlowitz et al. found survival benefits of 11–15.5 months in both bulbar and non-bulbar ALS patients (129). Using NIV ≥4 h were associated with longer survival for patients with ALS (134). NIV was well-tolerated and may improve quality of life and survival in patients with Duchenne muscular dystrophy (DMD) (135, 136) and SMA (137).

#### Polysomnography (PSG) with capnometry (“Gold Standard”) plays a role in determining the nature and severity of SDB in NMD

SDB in NMD patients includes pseudo-central or diaphragmatic SDB, central sleep apnea, obstructive sleep apnea, nocturnal hypoxemia, and hypoventilation in sleep. Non-invasive ventilation is indicated at the first sign of hypoventilation. A sleep study should be considered with at least one symptom and/or sign related to respiratory muscle weakness such as dyspnea, tachypnea, orthopnea, disturbed sleep, morning headaches, accessory muscle use at rest, paradoxical breathing, daytime fatigue, or daytime sleepiness (ESS > 9) (138). A PSG (“**Gold Standard**”) can evaluate respiratory muscle weakness during sleep and confirm the need for NIV. The absence of nocturnal hypoxemia does not exclude nocturnal hypercapnia. Nocturnal hypercapnia predicts impending daytime hypercapnia and is an indicator for nocturnal NIV before daytime hypercapnia occurs (139).

Scoring respiratory events during sleep in patients with NMD requires specific expertise. Patients with NMD usually present a decrease in airflow and respiratory effort with or without a reduction in pulse oximetry (SpO_2_). These events may be associated with increased transcutaneous carbon dioxide (TcCO_2_). Paradoxical breathing may result from respiratory muscle weakness and should not be interpreted as “obstructive events” (126). Periods of “reduced ventilation” or paradoxical breathing, especially during REM sleep, justify the initiation of NIV in patients with NMD (139).

According to the 2017 AASM guidelines, an unattended sleep study (“**Silver Standard**”) is not recommended in adults with NMD or known or suspected hypoventilation (140). However, as technology advances, “Silver Standard” sleep study devices that can accurately determine sleep stages plus capnography may be used to evaluate SDB in patients with NMD. The integrated Mobile Sleep Medicine Unit is designed to overcome the shortcomings of the traditional sleep medicine testing model and can deliver the entire spectrum of sleep testing to individuals with NMD. The iMSM includes sleep testing and PAP titration technology, which can be delivered to all levels of patient care from hospital to home, under the supervision of a board-certified sleep medicine physician. It means that we can deliver the Mobile Sleep Unit with portable PSG, capnography, and PAP titration directly to a patient's home and provide virtual monitoring for ventilators if necessary.

#### In-lab titration of NIV by PSG is not required but recommended by AASM before initiating NIV to identify optimal settings, reduce patient-ventilator asynchrony and improve treatment tolerance

Guidelines to initiate ventilation in NMD defined in 1999 include the presence of symptomatic daytime hypercapnia (PaCO_2_ ≥ 45 mmHg), nocturnal desaturation (SpO_2_ ≤ 88% for 5 consecutive minutes), MIP < −60 cmH_2_O, or FVC < 50% predicted (141). Commonly used NIV modes include Bilevel PAP with a backup rate (BPAP ST), or volume assured pressure support (VAPS) (66). Titration of non-invasive ventilation by attended polysomnography is the gold standard to identify effective parameters to correct SDB, including hypoventilation as well as central, pseudo-central, and obstructive sleep apnea. Patient-ventilator desynchrony may include ineffective effort, triggering asynchrony, cycling asynchrony, etc (142, 143). An in-lab titration of NIV by polysomnography study may improve patients' NIV treatment tolerance by observing and reducing patient-ventilator asynchrony (142).

The AASM consensus in 2010 non-invasive positive pressure ventilation (NPPV) titration with PSG to determine an effective level of nocturnal ventilatory support in patients with chronic alveolar hypoventilation. If the patient is started on NPPV empirically, a PSG would be considered necessary to confirm that the final settings are effective and to identify desynchronization or arousals from leaks (144).

However, logistic issues prevent attended sleep studies from being utilized in patients with NMD. These issues include long wait times, mobility, transportation, lift use, insurance coverage, etc.

#### Telemonitoring is one of the most revolutionary changes in home-assisted ventilation

Evidence is emerging that telehealth provides timely and cost-effective support for an individual with motor neuron disease (MND). Respiratory assist devices (RAD) and portable home ventilators transmit patient usage and home ventilation machines' efficacy data to a cloud-based platform *via* wireless networks. These enable physicians to access efficacy data updated daily *via* a cloud-based platform to provide personalized ventilation machine management at home and individualized care. Physicians may view breath-by-breath waveforms, trends in clinical data, and precise therapy values over periods of days, weeks, and months within 1 to 24-h windows.

Through telemonitoring, physicians can monitor disease progression, titrate modes and settings in a monitored environment and in a stepwise fashion, depending on the patient's symptoms and tolerance. In addition to optimizing device function, clinicians can quickly identify and troubleshoot ventilation issues.

Studies have shown that patients with ALS can be well-managed at home *via* telemonitoring. Benefits of telemonitoring include reduced ED visits and hospital admissions, increased survival, and improved functional status in patients with ALS (145, 146).

### The integrated mobile sleep medicine care model

The integrated **Mobile Sleep Medicine Care Model (iMSM)** is an innovative, progressive, patient-centered, integrative, complete cycle, clinical-outcome-based program that uses a Mobile Sleep Medicine Unit as a methodological tool—a “sleep lab on wheels”—designed to improve systematic access to OSA management and sleep care for approximately 80% of Americans at all healthcare levels, from hospital to home (67).

**Figure 1:**
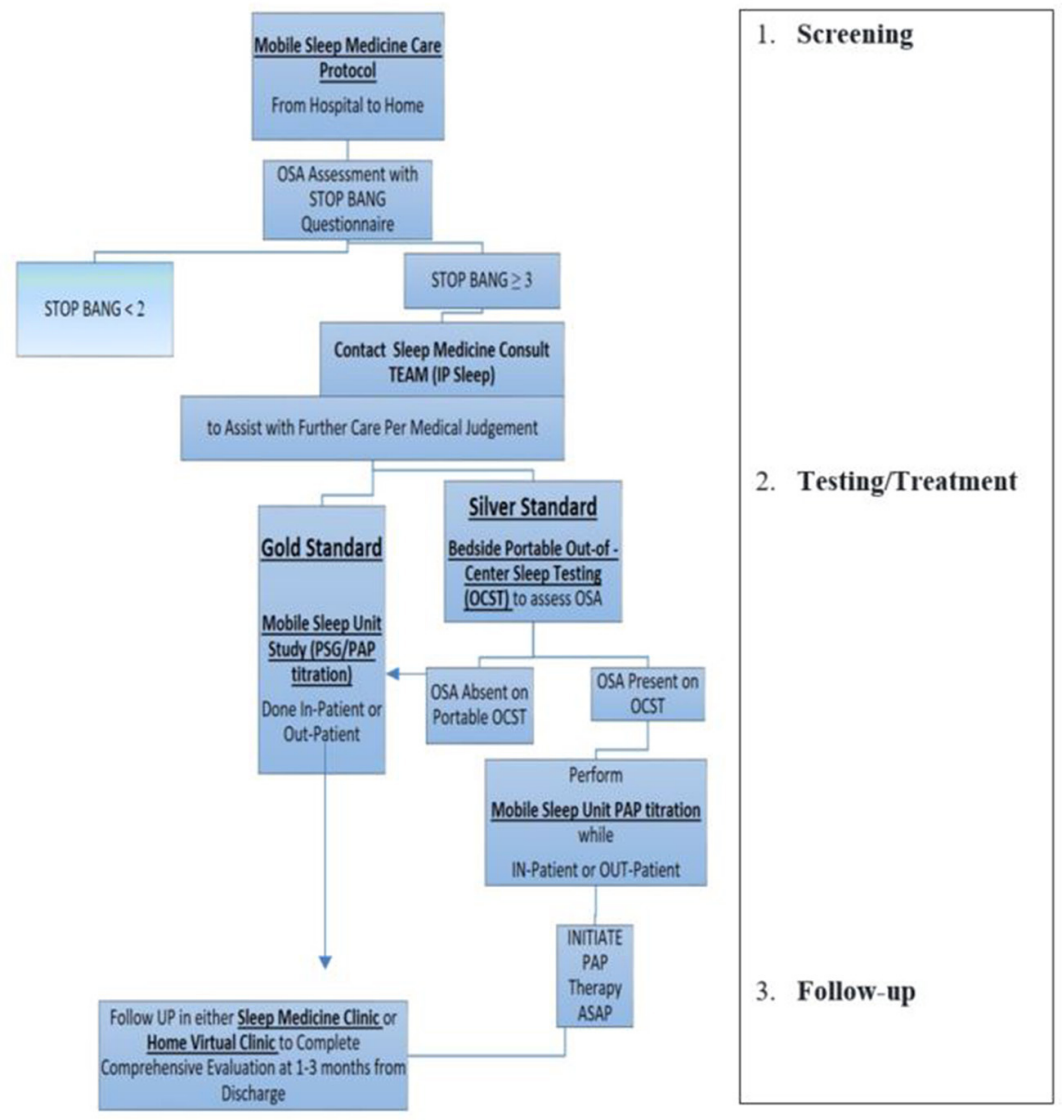
Example of mobile sleep medicine care protocol. (1) *Screening:* Designed to provide effective and efficient SDB screening for patients admitted to hospital (or any other health care facility, e.g., rehab, specialty/subspecialty clinic, etc.), including patients with a high risk of CCV (stroke, MI, CHF, atrial fibrillation, hypertension, preeclampsia, etc.). After completing SDB screening either in-person, *via* EMR, or *via* Tele-Virtual/My Chart system, the Primary Team communicates with the Mobile Sleep Medicine Team/ in-patient Sleep Medicine Consulting Team (IP Sleep) to request sleep consultation for further management. (2) *Testing/Treatment:* The board-certified sleep medicine physician from the integrated mobile sleep medicine Team will discuss the sleep study results with patients and coordinate further management. Designed to deliver timely sleep medicine expertise to patients who screen positive for OSA. The IP sleep/mobile sleep medicine team evaluates patients and develops the diagnostic and treatment plan using mobile sleep unit technology. (3) *Follow-up:* IP sleep/mobile sleep medicine team coordinates further sleep medicine care in a sleep clinic, sleep lab, home health, and virtual telemedicine health. https://d11tooehygcg9z.cloudfront.net/My.AASM/AgentsOfChange/587417.pdf; https://aasm.org/winners-of-inaugural-change-agents-competition-propose-new-approaches-for-sleep-apnea-care.

The protocol for the iMSM delivery model includes three levels of operations (see Figure 1):

1) Screening.
2) Testing/Treatment.
3) Follow-up.

### Components of the mobile sleep medicine unit

➢ *Module 1*. “Gold Standard”: Mobile Polysomnography (mPSG).
➢ *Module 2*. “Silver Standard”: Portable Out-of-Center Sleep Testing (OCST) (Nox-T3, Embletta, Stardust, ApneaLink Air, MediByte Jr, Alice, Cerebra Sleep System, BresoDx, etc.).

• Synonyms:

– Cardiopulmonary studies (CP).
– hospital/home sleep ambulatory testing (HSAT).
– (OCST) = (CP) = HSAT.

➢ *Module 3*. PAP titration unit: CPAP/BiPAP/AVAPS/ASV.

**Mobile Sleep Unit—“sleep lab on wheels”—**is a technological, methodological tool designed to deliver sleep medicine evaluation directly to the patient's bedside. The mobile sleep unit assembles with three functional technological modules representing modern sleep technology, which is the standard of care in the sleep medicine (67).

### Mobile sleep medicine unit

*Module 1: “Gold Standard”*: Mobile PSG-attended type 1 sleep testing device.

- Mobility and portability: Full sleep lab service delivered directly to the patient's bedside, either inside the hospital or at the patient's place of residence.

*Module 2: “Silver Standard”*: Unattended type 3 sleep testing device named in sleep literature (detailed above).

- Mobility and portability: Type 3 (four channels) sleep lab service is delivered directly to the patient's bedside, either inside the hospital or at the patient's place of residence, with an average set-up time of 5–10 mins.

*Module 3: PAP titration unit: CPAP/BiPAP/AVAPS/ASV:* Provides titration sleep study “on the spot.”

- Used for PAP titration in combination with Module 1/mobile PSG (“Gold Standard”) to perform mobile-attended PSG/Split or/PAP titration studies.

### Cost: Vertical integration payment system (VIPS) model

Designed to obtain collections from sleep-related operations performed at each level of care from hospital to sleep lab/home:

1) Technical charges and professional charges will be collected based on appropriate CTP codes for the type of study performed.
2) Sleep studies (unattended and attended).
3) Clinical in-patient sleep consults and outpatient sleep clinical visits.

a. Collections from each level will be combined to 100% and directed to support mobile sleep unit operations from hospital to home.
b. The sources of reimbursement obtained from each level of care.

a) *Acute hospital level of care*

i. Screening/evaluation for SDB will bring up diagnosis-related group reimbursement based on the following.
ii. Elevated complexity of comorbidities rule and increased case mix index.
iii. Improvement of hospital performance statistics due to a significant reduction in readmission rates in sensitive CCV population (CHF, chronic respiratory failure, obesity, hypoventilation, stroke, atrial fibrillation, MI, hypertension, seizure disorders, myasthenia gravis, ALS, etc.). All admitted patients in the CCV population are at high risk for SDB and should be evaluated by the inpatient mobile sleep medicine team (IP Sleep Team). The IP sleep team would decide the appropriate delivery (i.e., Gold vs. Silver Standard) for a given patient based on local standards of care and the availability of devices in the community. The mobile sleep medicine model provides flexibility in diagnosing and delivering necessary sleep care without delay.
iv. 1–33% of collections from each level will be directed to support mobile sleep unit operations from hospital to home. These are approximate, proposed figures, which can be modified based on local administrative protocols for utilization of services. Each given IP sleep team would choose its own proportion of collections to fit its needs for sustained clinical operations and to make the service self-sufficient based on local finances, insurance coverage, administrative support, grants, etc.

b) *Sleep lab/sleep center:* Sleep medicine referrals from inpatient sleep medicine consulting service (IP sleep):

- Will boost the sleep referral base to the internal sleep lab/center by about 1,300 patients per year (5 consults per day × 5 days per week × 52 weeks).
- Will prevent “referral leaks” to external systems and maintain the integrity of collections.
- 1–33% of collections from each level will be directed to support Mobile Sleep Unit Operations from hospital to home.

c) *Home health integration:* Patient-centered, unattended, or/and attended sleep studies will be conducted at the patient's residence (house, rehab, nursing home, etc.) and achieve the following:

- Integrate mobile sleep medicine care into the home health care model.
- Reduce the operational costs by 50% with a flexible technician: patient ratio (from 1:3 to 1:10) for unattended studies with strong reimbursement for each study performed.
- 1–33% of collections from each level will be directed to support mobile sleep unit operations from hospital to home.

The *mobile sleep medicine care model* prioritizes effective, efficient, and patient-centered sleep medicine care; therefore, all parties and segments of care that receive and provide clinical sleep medicine services will benefit in various measurable ways (67).

A. *Benefits for patients*

1) Patient-centered sleep care is delivered directly and conveniently to a patient at any setting/level of care: hospital, home, virtual telemedicine clinic, or in-person clinic.
2) The *iMSM* improves the overall patient experience by bringing sleep medicine expertise and testing directly to the patient's bedside without the unnecessary delays in care currently observed in the traditional sleep medicine model (e.g., self-scheduling for sleep clinic and sleep lab with long waiting times, and/or multiple missed/canceled appointments, etc.).
3) Improved sleep quality, sleep-related quality of life, participation in recovery and rehab activities due to controlled OSA-related issues: daytime sleepiness, fatigue, concentration and/or memory, altered mental status, delirium, and dyspnea.

B. *Benefits for Hospital/Healthcare System*

1) Screening/evaluation for SDB will upgrade the case-mix index and bring up diagnosis-related group reimbursement based on:

a) Elevated complexity of comorbidities rule and increased case mix index.
b) Improvement of hospital performance statistics due to:

i. Significant reduction in in-hospital mortality and readmission rates in CCV-sensitive populations (CHF, chronic respiratory failure, obesity hypoventilation, Stroke, atrial fibrillation, MI, hypertension, seizure disorders, neuromuscular disease, myasthenia gravis, ALS, etc.).
ii. Shortened the length of the following:

a. The intensive care unit stays.
b. Hospital stays (i.e., patients with OSA, OHS, post-operative respiratory failure, COPD, CHF, etc.).

iii. Prevention of escalation in the level of care, intubation, transfer to the intensive care unit, a rapid response team.
iv. Prevention of hypoxemia and/or hypoventilation.
v. Control of referral base and prevention of referral leaks (increase referral base to sleep center by 1300 patients per year).

C. *Benefits for payors*

1) Low cost for improved access to Neuro-Sleep Medicine care for pediatric and adult patients.
2) Reduced costs and more effective management of CCV, AD, and NMD.

D. *Benefits for the Academic Neuro-Sleep Medicine Field:*

1) Expansion of neurosomnology into all medical settings and levels of care with opportunities to monitor relevant SDB-related clinical outcomes and measure responses to targeted clinical interventions in real time while a patient is moving *via* levels of care;
2) Establishing the methodological basis for the development of digital evidence-based precision neuro-sleep medicine:

a) To effectively control and manage pertinent comorbidities in CCV, AD, and NMD.
b) Improve recovery and rehabilitation.

3) Removal of the “stigma” of being an “Outpatient Only” specialty with minimal impact on patient's clinical outcomes.

E. *National health benefits*

1) Overall reduction of SDB-related morbidity and mortality.
2) Data from iMSM would contribute to the development of evidence-based precision sleep medicine.

The **integrated iMSM, if implemented in the** general neurological practice, would provide the systematic screening, testing, and best-practice management of the most common sleep-related breathing disorders: OSA, HHS and potentially other sleep disorders.

We anticipate that **iMSM** with **mobile sleep unit methodology** has the potential for a positive, measurable impact on all parties involved in neurological care, including the neurological patient, the hospital/healthcare system, and the fields of neurology and sleep medicine.

## Author's note

The mobile sleep medicine model was presented during the 2021 AASM Sleep Disruptors competition and won the People's Choice Award. Information can be found at the following links: https://d11tooehygcg9z.cloudfront.net/My.AASM/AgentsOfChange/587417.pdf; https://aasm.org/winners-of-inaugural-change-agents-competition-propose-new-approaches-for-sleep-apnea-care.

## Author contributions

All authors listed have made a substantial, direct, and intellectual contribution to the work and approved it for publication.

## Funding

MB has received in-kind support for his research program from Braebon Medical Corporation, Interaxon, ResMed, and BresoTec. The funders were not involved in the study design, collection, analysis, interpretation of data, the writing of this article, or the decision to submit it for publication.

## Conflict of interest

The authors declare that the research was conducted in the absence of any commercial or financial relationships that could be construed as a potential conflict of interest.

## Publisher's note

All claims expressed in this article are solely those of the authors and do not necessarily represent those of their affiliated organizations, or those of the publisher, the editors and the reviewers. Any product that may be evaluated in this article, or claim that may be made by its manufacturer, is not guaranteed or endorsed by the publisher.
